# White spot syndrome virus (WSSV) modulates lipid metabolism in white shrimp

**DOI:** 10.1038/s42003-023-04924-w

**Published:** 2023-05-20

**Authors:** Yen Siong Ng, Cheng-Shun Cheng, Masahiro Ando, Yi-Ting Tseng, Shu-Ting He, Chun-Yuan Li, Shu-Wen Cheng, Yi-Min Chen, Ramya Kumar, Chun-Hung Liu, Haruko Takeyama, Ikuo Hirono, Han-Ching Wang

**Affiliations:** 1 Department of Biotechnology and Bioindustry Sciences, College of Bioscience and Biotechnology, National Cheng Kung University, Tainan, Taiwan; 2 Research Organization for Nano and Life Innovations, Waseda University, Tokyo, Japan; 3 International Center for the Scientific Development of Shrimp Aquaculture, National Cheng Kung University, Tainan, Taiwan; 4 Department of Aquaculture, National Pingtung University of Science and Technology, Pingtung, Taiwan; 5 Department of Life Science and Medical Bioscience, Waseda University, Tokyo, Japan; 6 Computational Bio Big-Data Open Innovation Laboratory (CBBD-OIL), National Institute of Advanced Industrial Science and Technology, Tokyo, Japan; 7 Institute for Advanced Research of Biosystem Dynamics, Waseda Research Institute for Science and Engineering, Tokyo, Japan; 8 Department of Marine Biosciences, Tokyo University of Marine Science and Technology, Tokyo, Japan

**Keywords:** Virus-host interactions, Viral host response, Viral pathogenesis

## Abstract

In addition to the Warburg effect, which increases the availability of energy and biosynthetic building blocks in WSSV-infected shrimp, WSSV also induces both lipolysis at the viral genome replication stage (12 hpi) to provide material and energy for the virus replication, and lipogenesis at the viral late stage (24 hpi) to complete virus morphogenesis by supplying particular species of long-chain fatty acids (LCFAs). Here, we further show that WSSV causes a reduction in lipid droplets (LDs) in hemocytes at the viral genome replication stage, and an increase in LDs in the nuclei of WSSV-infected hemocytes at the viral late stage. In the hepatopancreas, lipolysis is triggered by WSSV infection, and this leads to fatty acids being released into the hemolymph. β-oxidation inhibition experiment reveals that the fatty acids generated by WSSV-induced lipolysis can be diverted into β-oxidation for energy production. At the viral late stage, WSSV infection leads to lipogenesis in both the stomach and hepatopancreas, suggesting that fatty acids are in high demand at this stage for virion morphogenesis. Our results demonstrate that WSSV modulates lipid metabolism specifically at different stages to facilitate its replication.

## Abstract (web summary)

Investigation into the lipid metabolism shifts occurring in shrimp infected with white spot syndrome virus provides insight into how this virus modulates host metabolism in a way that creates a favorable environment for its replication.

## Introduction

By inducing metabolic reprogramming, cancer cells are able to meet their demand for metabolic intermediates and energy^1–3^. Subsequently, to facilitate rapid cancer cell growth and proliferation, biomolecules such as nucleic acids, proteins and lipids are generated^4^. An important part of this metabolic reprogramming is the altered lipid metabolism that is observed in many different cancer cell types^5,6^. Lipids are versatile, and in cancer cells they can play several roles. Thus for example the fatty acids (FAs) in cancer cells can be used to construct biological membrane during cancer cell proliferation^7,8^. Enzymes involved in fatty acid oxidation (FAO) are also often overexpressed in cancer cells to accommodate the production of energy and NADPH^9,10^. Protein modification by lipid moieties is also associated with cancer metabolism^11,12^.

As lipids are highly involved in many biological processes in a cancer cell, they also participate in the various mechanisms of viral replication, ranging from entry to release^13,14^. Lipid metabolic reprogramming has been found in several viruses which trigger the Warburg effect: Kaposi’s sarcoma herpesvirus (KSHV)^15^, hepatitis C virus (HCV)^16^, and human papillomavirus (HPV)^17^. By changing the host’s lipid profile, the dengue virus (DENV) induces membrane alteration, thereby promoting viral replication and protecting the virus from antiviral defense mechanisms^18^. DENV also makes use of the lipid droplet protein AUP1 to initiate lipophagy, which in turn generates phospholipids to facilitate virus replication^19^. A KSHV tegument protein targets lipid rafts of host cell membrane to facilitate the release of viral particles^20^, while a protein from HCV called NS5A contributes to the increased size of lipid droplets and promotes the HCV morphogenesis process^21^. Meanwhile, in enveloped viruses, it is common for lipids from the host cell membrane to be adapted for use as the virus outer coat^22^. All of these examples demonstrate how modification or alteration of lipid metabolism by a virus is required to facilitate production of the infectious virion particles.

In line with other viruses, a deadly shrimp virus called white spot syndrome virus (WSSV) has been shown to change the population and composition of various fatty acid species during infection: at the viral genome replication stage (12 hpi), WSSV induces lipolysis, in which the fatty acids undergo β-oxidation to generate energy and biomolecules, while at the late stage (24 hpi), WSSV enhances the expression of fatty acid synthase (FAS) to support lipogenesis, resulting in the production of large quantities of fatty acid for virion morphogenesis^23^. In the present study, we further investigate the mechanisms behind these lipid metabolism shifts in WSSV-infected shrimp. We first use fluorescent staining to investigate the localization of lipid droplets in infected hemocytes at different stages of virus replication. Next, we measure the activity of lipase, a key enzyme in lipolysis, and monitor its effect on the amount of fatty acids in shrimp tissue. To investigate how β-oxidation affects energy production and virion morphogenesis, we then used Etomoxir, a CPT1 inhibitor, to block β-oxidation in WSSV-infected shrimp. Finally, we block both β-oxidation and fatty acid synthesis in infected shrimp and observe how this impacts the species of long-chain fatty acids (LCFAs) in shrimp tissue.

## Results

### WSSV leads to the enhancement of lipid droplets in the nuclei of shrimp hemocytes at the late stage of virus replication

**Fig. 1:**
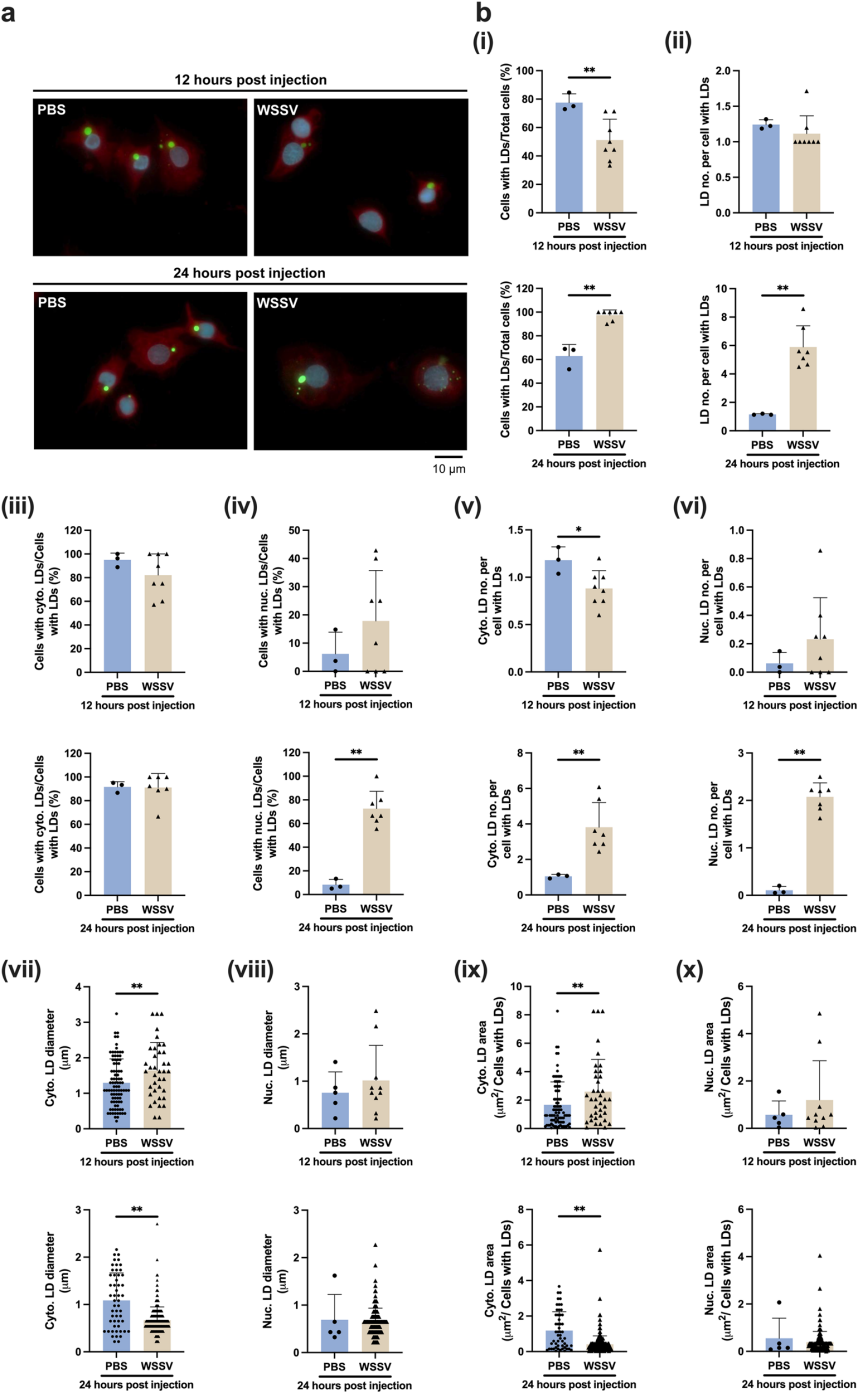
Lipid droplets in WSSV-infected shrimp hemocytes. **a** Hemocytes collected from WSSV-infected shrimps and PBS-injected shrimps (Control) at 12 hpi and 24 hpi were stained with DAPI, Evan’s blue and BODIPY for visualizing nucleus, cytoplasm and lipid droplets (LDs), respectively. The scale bar represents 10 μm. **b** Various metrics were used to quantify the LDs: [i] Percentage of cells with detected LDs; [ii] Number of LDs puncta per cell with LDs; [iii] Percentage of cells with cytoplasmic LDs/total cells with LDs; [iv] Percentage of cells with nuclear LDs/total cells with LDs; Number of LD puncta present in [v] cytoplasm and [vi] nucleus per LD-harboring hemocyte; Maximal LD diameter in [vii] cytoplasm and [viii] nucleus; LDs positive area in [ix] cytoplasm and [x] nucleus. Results were expressed as the mean (*n* > 3) of the proportion of permeabilized cells relative to the total number of specified denominator cell type (*n* > 100). Each bar represents mean ± SD. Asterisks indicate differences between WSSV and PBS groups (*p < 0.05, **p < 0.01).

We previously showed that WSSV alters the variety of long-chain fatty acids during infection^23^. Here, in order to further understand the dynamics of lipid accumulation in WSSV-infected shrimp, we used BODIPY 493/503 staining to monitor changes in the number of lipid droplets, their cellular distribution, and droplet sizes at both 12 and 24 hpi (Fig. 1a). At the viral genome replication stage (12 hpi), fewer cells in the WSSV-infected group contained lipid droplets (LDs) compared to the PBS controls (Fig. 1bi), suggesting the occurrence of lipolysis at this stage. Conversely, at the viral late stage (24 hpi), the number of hemocytes harboring LDs was significantly increased in the WSSV infection group, and each hemocyte with LDs also contained more LD puncta (Fig. 1bi, ii). WSSV did not affect the number of hemocytes harboring cytoplasmic LDs at either stage (Fig. 1biii), but at the late stage, the WSSV-infected shrimp had more hemocytes with LDs in the nucleus (Fig. 1biv). WSSV also increased the number of both cytoplasmic and nuclear LDs at the late stage (Fig. 1bv, vi). The increased number of LDs in the hemocytes of the WSSV-infected group suggested that WSSV might trigger lipogenesis at the late stage.

In the WSSV group, the diameter and total cross-sectional area of the cytoplasmic LDs were significantly increased at the viral genome replication stage and decreased at the viral late stage (Fig. 1bvii, ix). However, these changes were not observed in the nuclear LDs (Fig. 1bviii, x).

### WSSV enhances lipase activity in hepatopancreas and increases free fatty acids (FFAs) in hemocytes and hemolymph

**Fig. 2:**
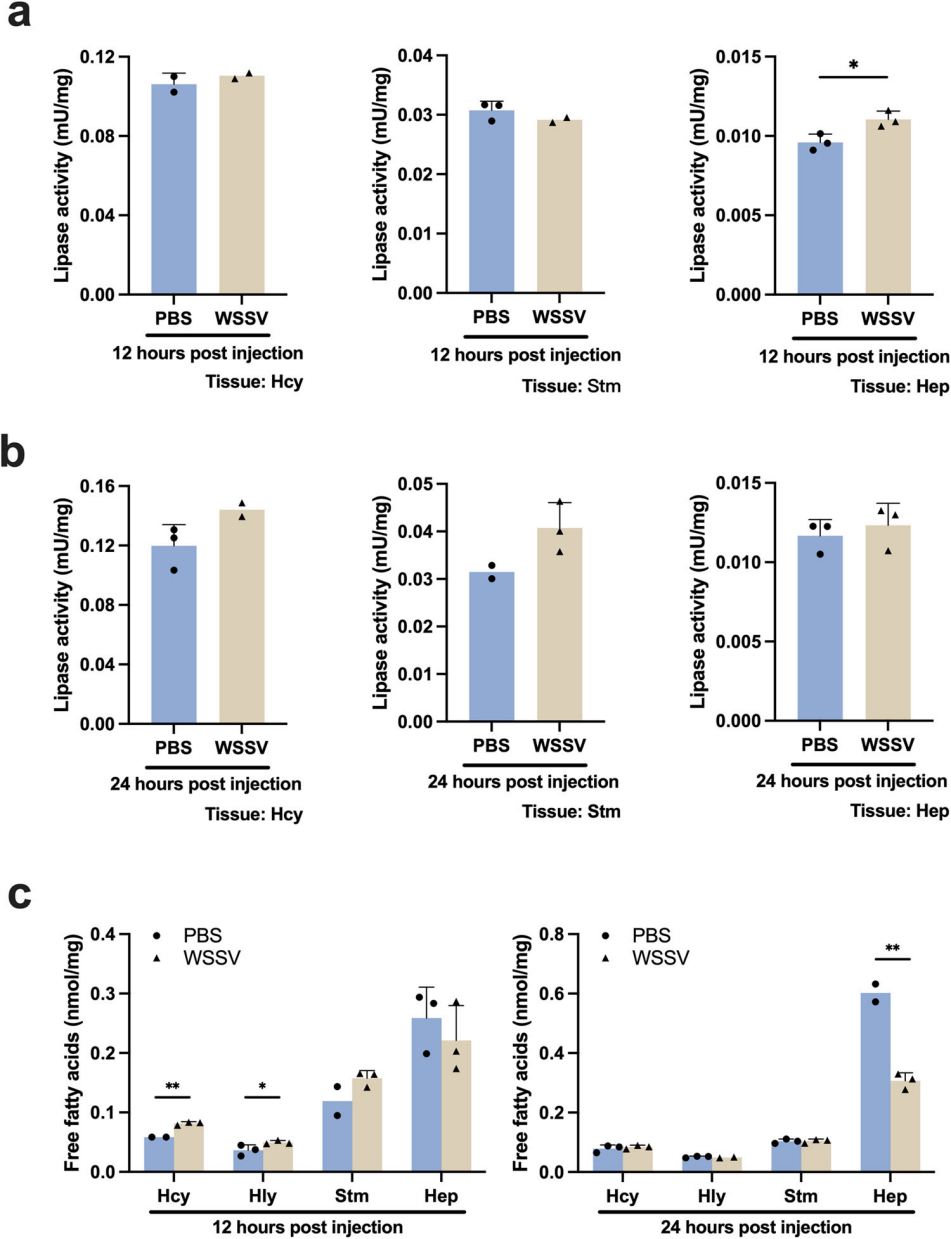
Lipase activity and free fatty acids in various tissues of WSSV-infected shrimp. The indicated tissues of PBS-injected and WSSV-injected shrimp were collected at 12 hpi and 24 hpi and subjected to **a**, **b** lipase activity measurement and (**c**) free fatty acid quantification. Lipase activity was increased in infected hepatopancreas at 12 hpi. Free fatty acids were increased in hemocytes and hemolymph at 12 hpi, whereas a significant decrease of free fatty acids was found in infected hepatopancreas at 24 hpi. Each bar represents mean ± SD, *n* = 2 ~ 3 pool samples (3 shrimp per pool). Asterisks indicate differences between WSSV and PBS groups (*p < 0.05, **p < 0.01). Hcy hemocytes, Hly hemolymph, Stm stomach, Hep hepatopancreas.

We examined the lipase activity in three different tissues of WSSV-infected shrimp. At the viral genome replication stage, no difference in lipase activity was observed between the PBS and WSSV groups in hemocytes or stomach, but lipase activity was elevated in the WSSV-infected hepatopancreas (Fig. 2a). At the late stage, lipase activity was not changed in all WSSV-infected tissues (Fig. 2b). FFAs, which are produced by lipase activity, were also detected at higher levels in WSSV-infected hemocytes and hemolymph at the viral genome replication stage (Fig. 2c). This might be due to the elevated lipase activity in the hepatopancreas. Conversely, FFAs were significantly decreased in WSSV-infected hepatopancreas at the viral late stage (Fig. 2c), which might be a consequence of lipid depletion during the viral genome replication stage.

### β-oxidation is important for energy production in WSSV-infected shrimp

**Fig. 3:**
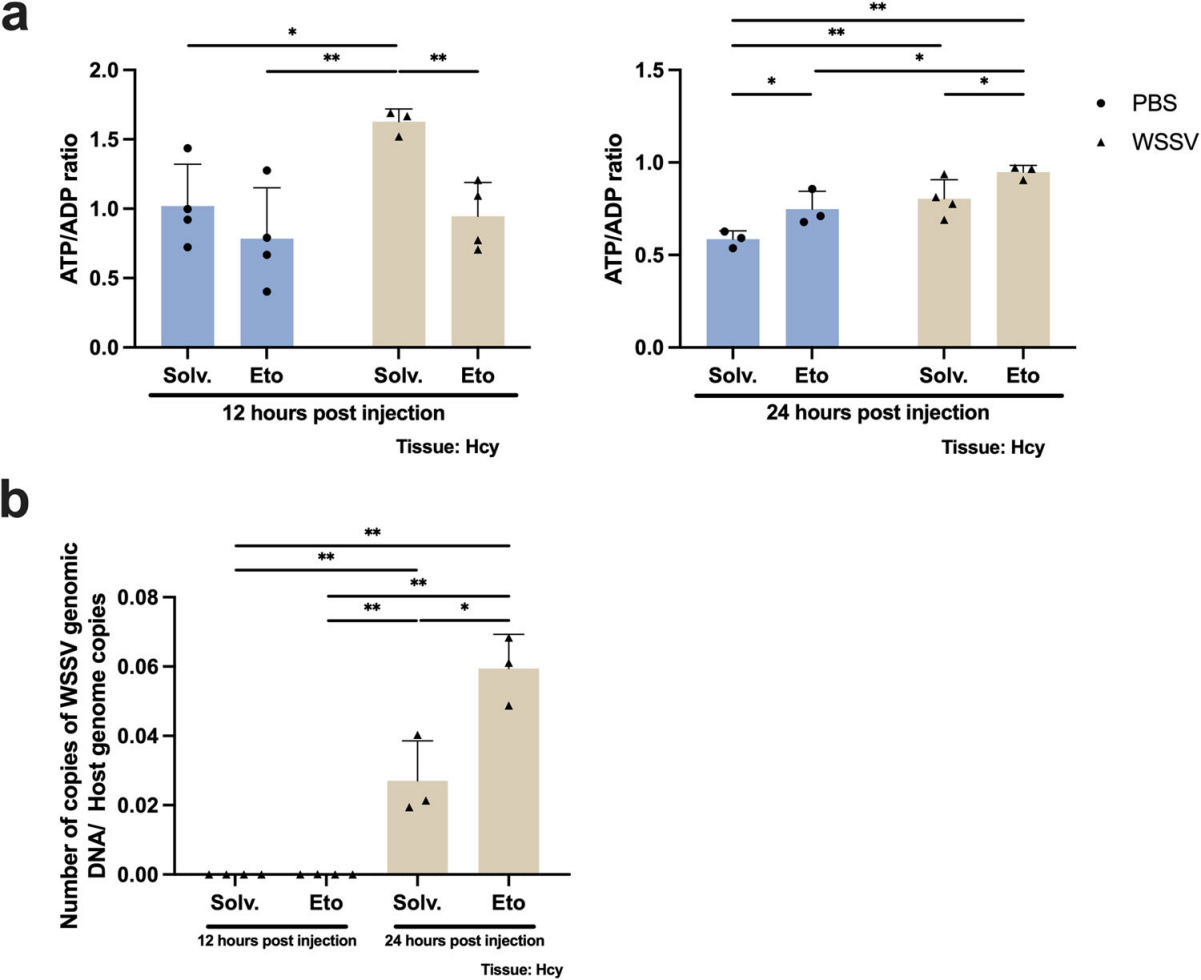
The effects of β-oxidation inhibition on energy production and viral DNA replication in WSSV-infected shrimp. Shrimp were injected with Etomoxir 10 h after WSSV challenge. **a** Hemocytes from 12 hpi and 24 hpi were subjected to an ATP/ADP ratio assay. Asterisks indicate differences between WSSV and PBS groups (*p < 0.05, **p < 0.01). **b** Hemocytes from both time points after WSSV challenge were used to quantify the viral genome copy numbers to assess viral DNA replication. Each bar represents mean ± SD, *n* = 3–4 pool samples (4 shrimp per pool). Asterisks indicate differences between WSSV groups (*p < 0.05, **p < 0.01). Solv. solvent, Eto etomoxir, Hcy hemocytes.

Under normal circumstances, lipid droplets and triglycerides are the substrates of lipase, and they can be degraded into FFAs which are then transported into the mitochondria and used to produce energy by β-oxidation^24,25^. Here, we treated shrimp with Etomoxir, a specific inhibitor of carnitine palmitoyltransferase I (CPT1), to block entry of the LCFAs into the mitochondria, thus inhibiting β-oxidation. When the β-oxidation was inhibited, we found that mitochondrial activity (as represented by the ATP/ADP ratio) in WSSV-infected hemocytes was significantly decreased at the viral genome replication stage but increased at the late stage (Fig. 3a). To determine whether β-oxidation is also important for virion production, we next measured the viral genome copy numbers in hemocytes and hemolymph. In hemocytes, viral genome copy number in the solvent group was increased at the viral late stage compared to the viral genome replication stage, while inhibiting β-oxidation led to even higher viral genome copy numbers at the late stage (Fig. 3b). Similarly, in the hemolymph, inhibiting β-oxidation resulted in significantly increased viral genome copy numbers at the late stage (Fig. S1). These results show that the inhibition of β-oxidation consistently leads to an increase in viral genomic DNAs in both hemocytes and hemolymph, and suggest that β-oxidation may be involved in WSSV replication.

### Long-chain fatty acids (LCFAs) are utilized via β-oxidation in WSSV-infected hepatopancreas

**Fig. 4:**
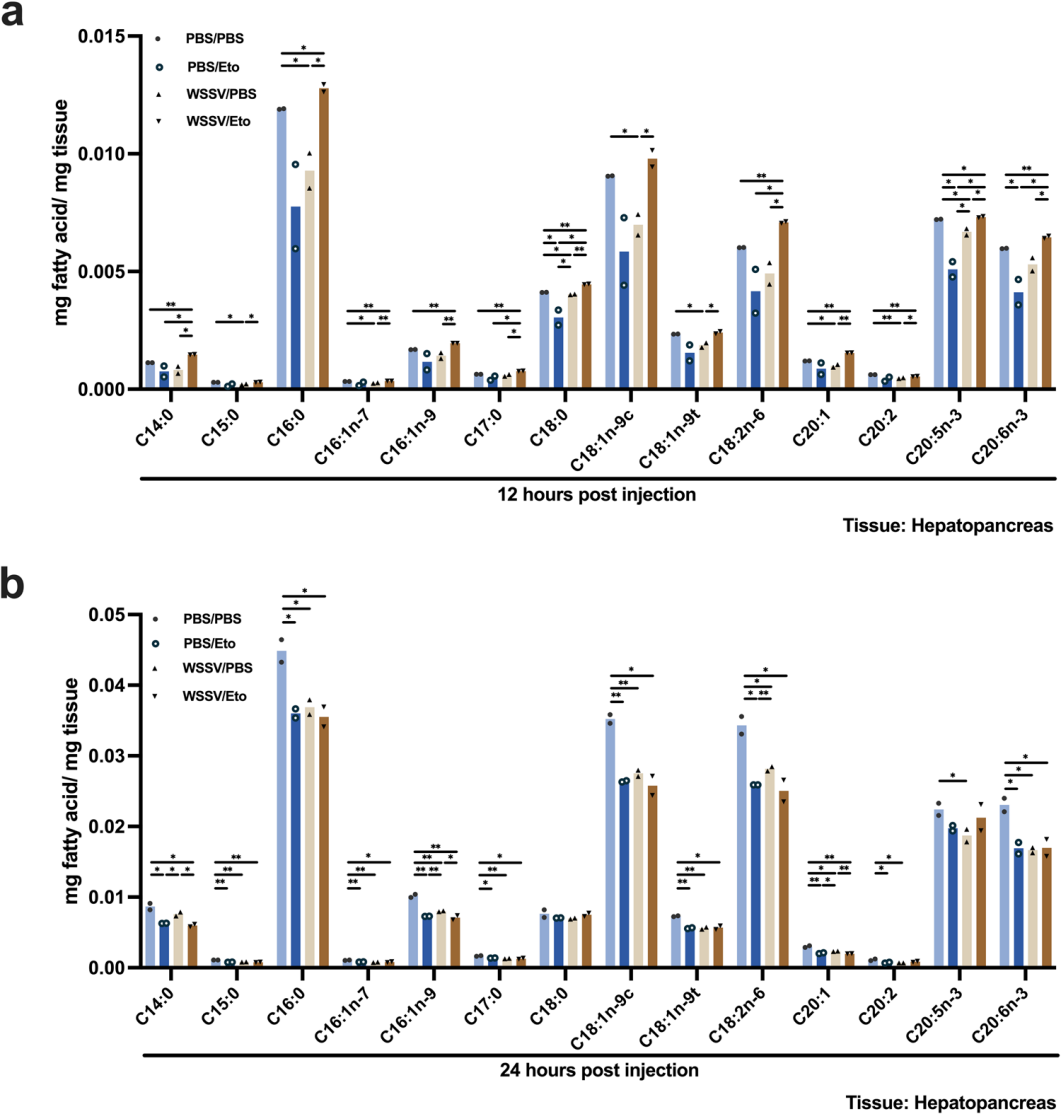
The effects of β-oxidation inhibition on LCFA profiles in WSSV-infected hepatopancreas. Hepatopancreas samples were collected from the WSSV-infected groups and PBS control groups at 12 and 24 hpi. The WSSV-infected groups were designated as WSSV/PBS and WSSV/Eto according to whether the WSSV-infected shrimps were treated with PBS or Etomoxir. Likewise, the PBS control groups were named PBS/PBS and PBS/Eto. LCFA profiles from **a** 12 and **b** 24 hpi were determined by gas chromatography mass spectrometry (GC/MS). The β-oxidation inhibition disrupted the WSSV-induced LCFA consumption at 12 hpi. Each bar represents mean ± SD, *n* = 2 pool samples (3 shrimp per pool). Asterisks indicate differences between WSSV and PBS groups (*p < 0.05, **p < 0.01).

Lipase was activated in the hepatopancreas at the viral genome replication stage (Fig. 2a), and we hypothesized that the LCFAs produced by the lipase activity might be shifted to β-oxidation. This would also be consistent with Hsieh et al.^23^, who reported that various LCFAs were decreased in infected stomach at the same stage. We therefore proceeded to quantify the amount of various LCFAs in the hepatopancreas under conditions of β-oxidation inhibition. At the viral genome replication stage, palmitic acid (C16:0), one of the most common saturated fatty acids found in animals, was significantly decreased by WSSV infection (WSSV/PBS group) compared to the PBS control (PBS/PBS group), but significantly increased when β-oxidation was blocked (WSSV/Eto group) (Fig. 4a). Similar results were also observed in other LCFAs such as C18:0, C20:1 and C20:2 (Fig. 4a), suggesting that, as expected, blocking β-oxidation prevented further metabolic processing of the LCFAs induced by WSSV. At the viral late stage, when lipolysis was no longer activated (Hsieh et al.^23^), inhibition of β-oxidation had no significant effect (Fig. 4b). This is consistent with the idea that the energy produced by β-oxidation is not required at this stage (Fig. 3a).

### Fatty acid synthase (FAS) is responsible for lipogenesis in WSSV-infected shrimp

**Fig. 5:**
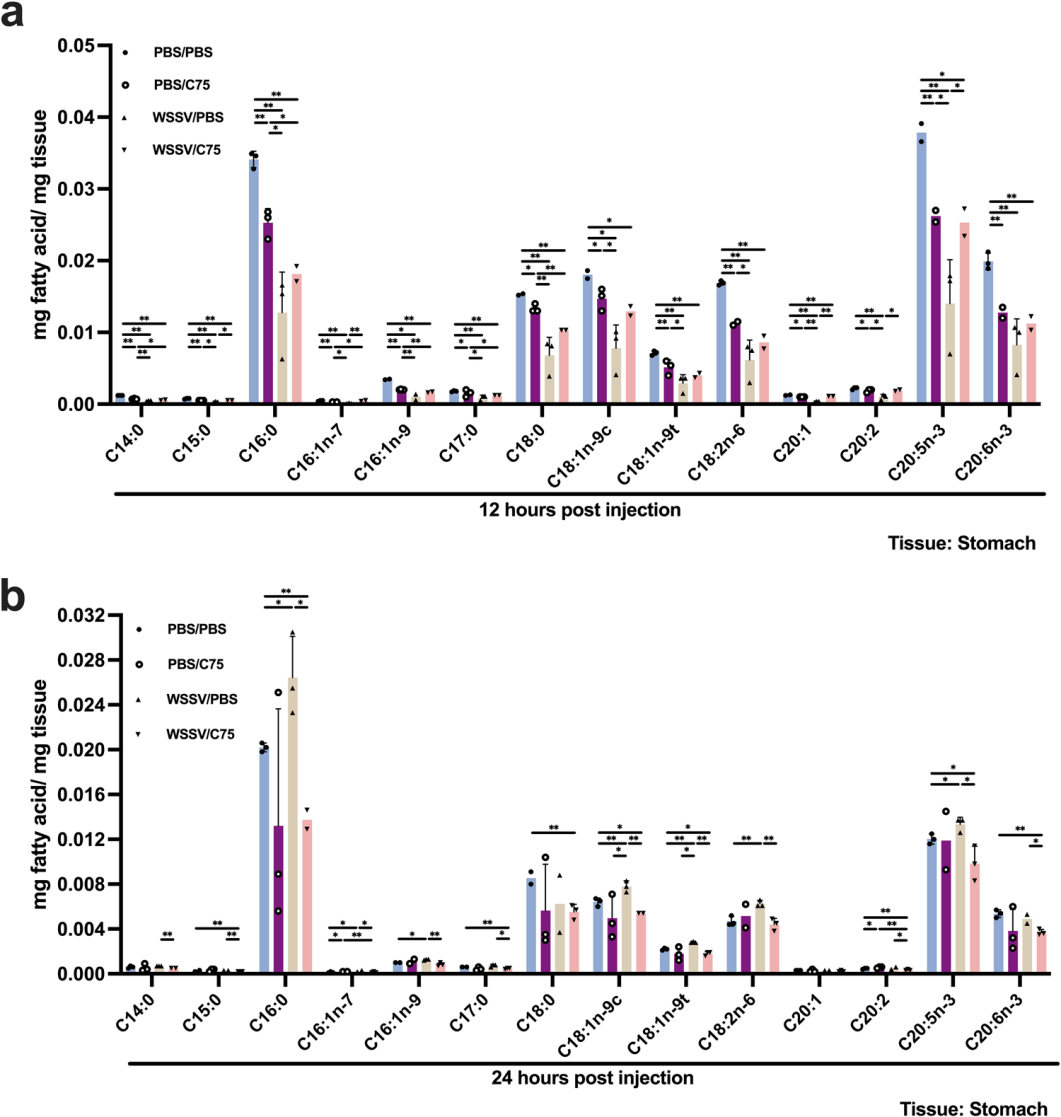
The effects of FAS inhibition on LCFAs profile in WSSV-infected stomach. C75 was injected into the shrimp 4 h after WSSV injection to inhibit FAS. The WSSV-infected groups were designated as WSSV/PBS and WSSV/C75 to denote WSSV-infected shrimp was injected with PBS or C75. PBS control groups were named as PBS/PBS and PBS/C75. Gas chromatography mass spectrometry (GC/MS) was employed to investigate the LCFAs profile in WSSV-infected stomach at **a** 12 hpi and **b** 24 hpi. FAS inhibition reduced production of the LCFAs at the late stage of infection. Each bar represents mean ± SD, *n* = 2–4 pool samples (3 shrimp per pool). Asterisks indicate differences between WSSV and PBS groups (*p < 0.05, **p < 0.01).

Hsieh et al.^23^ demonstrated the involvement of FAS in WSSV replication: 1. FAS expression was induced by WSSV through the PI3K-Akt-mTOR-HIF1α signaling pathway; 2. FAS was crucial for virion morphogenesis. To characterize the role of FAS in LCFA generation, at 4 h after injection with WSSV, the virus-challenged shrimp were intramuscularly injected with the FAS inhibitor C75, and the LCFAs were subsequently quantified in the stomach and hepatopancreas. In the stomach at the viral genome replication stage, various LCFAs were decreased by WSSV infection (WSSV/PBS group); however, with the exception of few LCFA, when FAS was also inhibited (WSSV/C75 group), then the magnitude of these decreases was reduced (Fig. 5a). At the late stage, several LCFAs, including C16:0, C18:1n-9c, C18:1n-9t, and C20:5n-3, were increased by WSSV infection (WSSV/PBS group), suggesting that lipogenesis was induced (Fig. 5b). However, FAS inhibition disrupted the generation of LCFAs in WSSV-infected shrimp (WSSV/C75 group) (Fig. 5b).

**Fig. 6:**
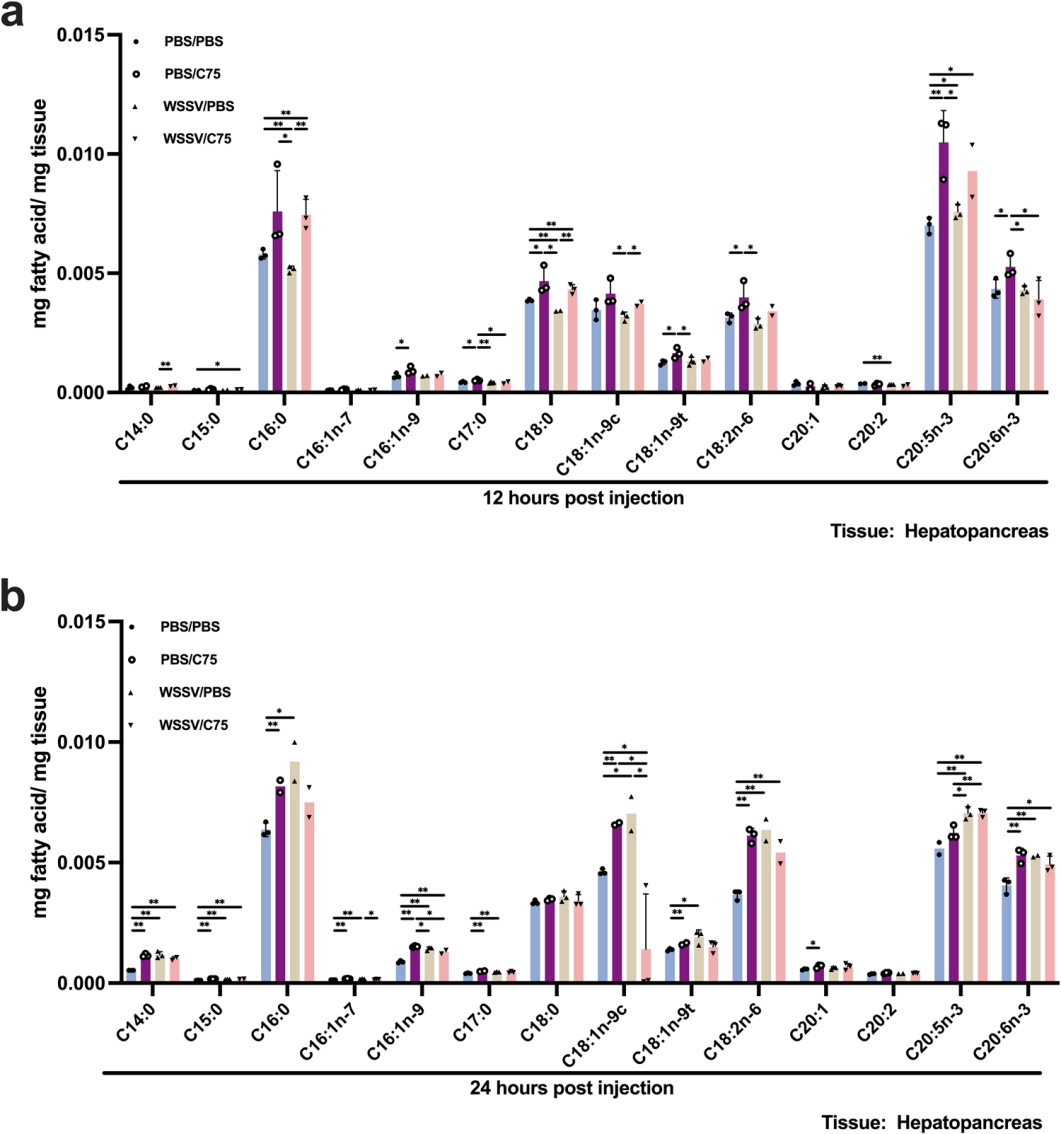
The effects of FAS inhibition on LCFAs profile in WSSV-infected hepatopancreas. Gas chromatography mass spectrometry (GC/MS) was employed to investigate FAS-inhibited WSSV-infected hepatopancreas at **a** 12 and **b** 24 hpi. The WSSV-infected groups were designated as WSSV/PBS and WSSV/C75 to denote WSSV-infected shrimp was injected with PBS or C75. PBS control groups were named as PBS/PBS and PBS/C75. Unlike the stomach (Fig. 5b), FAS inhibition had no effect on LCFA production in hepatopancreas at the late stage. Each bar represents mean ± SD, *n* = 2–4 pool samples (3 shrimp per pool). Asterisks indicate differences between WSSV and PBS groups (*p < 0.05, **p < 0.01).

As shown in Fig. 6a, WSSV also reduced the palmitic acid and stearic acid (C18:0) in infected hepatopancreas at the viral genome replication stage (WSSV/PBS group), but this reduction was canceled by FAS inhibition (WSSV/C75 group). At the late stage, various LCFAs were increased in infected hepatopancreas (WSSV/PBS group), while FAS inhibition had no significant effect (WSSV/C75 group) (Fig. 6b). These results are consistent with the Hsieh et al.^23^ report, in which lipolysis was triggered at the viral genome replication stage and lipogenesis at the late stage, and collectively they confirm that WSSV reroutes lipid metabolism in WSSV-infected shrimp.

## Discussion

Lipid metabolic reprogramming is found not only in cancer cells and cells infected by vertebrate viruses^26–28^, but also in virus-infected invertebrate cells^18^. Our present results, which include new data on the unbound fatty acids, now provide further details of how the lipid metabolism in WSSV-infected shrimp is rerouted. A previous study^23^, which found that lipolysis is induced at the viral genome replication stage whereas lipogenesis is induced at the late stage of virus replication, also reported that these metabolic shifts are characterized by changes in the profiles of the LCFAs, with several LCFAs being decreased at the viral genome replication stage. Our present LD staining results further show that there is a reduction in the LDs, especially the cytoplasmic LDs, at the viral genome replication stage (Fig. 1bi, v), thus providing further evidence that lipolysis is occurring at this stage. The FFAs released by this process then serve as a source for energy production via β-oxidation (Fig. 3a). Fatty acids are used in a similar way in a human cell line infected by dengue virus (DENV), where LDs are transported to lysosomes and the released fatty acids then undergo β-oxidation to generate ATP for virus replication^29^. As for the connection between β-oxidation and virion morphogenesis (Figs. 3b and S1), we hypothesize that the consumption of fatty acids via β-oxidation also generates the material required for the lipogenesis and virion morphogenesis that occurs at the late stage of viral replication. In support of this, we note that the number of hydrocarbon and cholesterol species are higher in the WSSV virion compared to the host-cell nuclei^30^, and that enterovirus redistributes host fatty acids to its replication compartments via lipolysis^31^, both of which observations are consistent with the idea that the consumed fatty acid might be repurposed for the production of specific lipids such as cholesterol. However, we also note that β-oxidation inhibition led to an increase in viral DNA both inside the hemocytes (Fig. 3b) and in the hemolymph (Fig. S1), which is hard to explain if β-oxidation really is driving virion morphogenesis. On the other hand, since our method for counting virion copy numbers would detect both complete and incomplete virions equally well, it is also possible that β-oxidation inhibition might be disrupting the process of virion morphogenesis in a way that causes large quantities of abnormal virions and/or naked viral DNA to be released into the hemolymph. Clearly, these possibilities will need to be further experimentally explored using a technique such as isotope tracing.

LD accumulation, which is observed in cells infected with HCV and SARS-CoV-2^32,33^, was also observed in the WSSV-infected hemocytes at the late stage of virus infection (Fig. 1bi, ii) as well as being reported by Hsieh et al.^23^. Here, most of the LDs were localized in the nuclei of infected hemocytes (Fig. 1biv, vi), which is where virion morphogenesis occurs^34,35^. Raman imaging also showed that lipid content was noticeably increased in the nucleus of infected hemocytes [at 60 hpi] (Fig. S2). To date, it remains unclear how WSSV might take advantage of the accumulated LDs localized in the hemocyte nucleus, but we speculate that they might provide a store of virus components to be used for virion morphogenesis, as they do for the hepatitis C virus, where the HCV nucleocapsid core and regulatory protein NS5A translocate from the endoplasmic reticulum (ER) to the LDs, which then act as a site for virion assembly^36^. Furthermore, the accumulated nuclear LDs in vertebrate cells may be involved in regulating gene expression that facilitates viral morphogenesis at the viral late stage^37^. The LDs might thus have two fates in the course of a WSSV infection: 1. at the viral genome replication stage, the LDs are broken down to produce energy and, possibly, to provide material for virion morphogenesis; 2. at the viral late stage, the LDs accumulate in the nucleus to support virion morphogenesis.

As an obligate parasite, viruses derive energy (in the form of ATP) from cellular hosts for their replication^38^. ATP/ADP ratio results shown in Fig. 3a show that WSSV elevated ATP in the solvent group at both stages (PBS control vs WSSV at 12 and 24 hpi). Similar results were reported by Apún-Molina et al.^39^, where a higher adenylic energy charge (AEC) was observed in WSSV-infected hepatopancreas at 24 hpi. This ATP elevation is consistent with previous research, which found that WSSV requires energy to facilitate its replication^39–41^. As discussed earlier, β-oxidation might be providing ATP for virus replication at the viral genome replication stage. Surprisingly however, at 24 hpi, β-oxidation inhibition resulted in elevated ATP levels regardless of infection status (Fig. 3a, Solvent group vs Etomoxir group at 24 hpi). We speculate that the long duration of β-oxidation inhibition might have led to the activation of other energy pathways in order to maintain cell viability.

Figure 2a shows that lipase activity was triggered in shrimp hepatopancreas, which is a lipid-storing tissue. Unlike the total fatty acids, which Hsieh et al.^23^ found to be decreased in the stomach at 12 hpi and increased at 24 hpi, what we found here suggests that at 12 hpi, FFAs might have been released from the hepatopancreas into the hemolymph and then taken up by hemocytes (Fig. 2c). We note that FA uptake is also promoted by other viruses for replication purposes^42–44^. Subsequently, the substantial reduction of FFAs in the hepatopancreas at the viral late stage (Fig. 2c) suggests that the hepatopancreas may act as a source of FAs during the virus infection. We further observed that several LCFAs were decreased in the WSSV-infected hepatopancreas at viral genome replication stage (Fig. 4a), but that when β-oxidation was inhibited, this WSSV-induced LCFA consumption was no longer observed (Fig. 4a). These results suggest that lipids stored in the hepatopancreas are broken down via lipolysis at the viral genome replication stage, and that the fatty acids produced by this process are then either subjected to β-oxidation or released directly into the hemolymph to fuel the WSSV-infected hemocytes. Conversely, at the viral late stage, inhibiting β-oxidation made no significant difference to the availability of the LCFAs (Fig. 4b), suggesting that at this time point, as proposed by Hsieh et al.^23^, the WSSV-induced lipid reprogramming shifted away from lipolysis and toward lipogenesis.

FAS, a major enzyme in lipogenesis, is activated in tumor cells for sustaining proliferation and in virus-infected cells for virion morphogenesis^45–47^. We previously found that FAS inhibition impairs virion morphogenesis but not viral protein expression^23^. Here we further found that the energy status of WSSV-infected hemocytes was not disrupted by FAS inhibition (Fig. S3). All these outcomes imply that FAS produces FA solely to be used in virus morphogenesis. As expected, at the viral late stage, FAS inhibition hampered WSSV-induced lipogenesis in the infected stomach (Fig. 5b); however, this effect was not seen in the hepatopancreas, except oleic acid (C18:1n-9c) (Fig. 6b). Surprisingly, FAS inhibition also retarded the consumption of some of the LCFAs in both WSSV-infected stomach and hepatopancreas (Figs. 5a and 6a).

Taken together, these results show how WSSV is able to modulate host lipid metabolism to establish a favorable environment for its replication: WSSV triggers either lipolysis or lipogenesis according to whether the virus is using the FAs as an energy source or as material for virus morphogenesis. This is the same kind of lipid reprogramming that is seen with some Flaviviruses^48–50^. In order to develop an effective antiviral strategy, it will be important to further elucidate the signaling pathways and viral proteins that support these mechanisms. While we have previously shown that WSSV induces FAS expression via the PI3K-Akt-mTOR-HIF1α pathway^23^, other signaling pathways might also be involved. It will also be interesting to investigate how WSSV is able to regulate the switch from lipolysis to lipogenesis at a specific time point after infection.

## Methods

### Experimental animals and virus inoculum

The white shrimp (*Litopenaeus vannamei*; 3 g body weight) used in this study were obtained from the Aquatic Animal Center at National Taiwan Ocean University, the International Center for the Scientific Development of Shrimp Aquaculture, National Cheng Kung University (NCKU) and the Department of Aquaculture, National Pingtung University of Science and Technology (NPUST). Shrimp were cultured in tank systems containing sterilized seawater (30 ppt; 25–27 °C) for 1–2 days before the experiments. The virus stock for the experimental inoculum was obtained by collecting hemolymph from moribund SPF (specific pathogen free) shrimp infected by the WSSV Taiwan isolate (GenBank Accession no. AF440570) and then diluting with phosphate buffer saline (PBS) (137 mM NaCl, 2.7 mM KCl, 10 mM Na_2_HPO_4_, 2 mM KH_2_PO_4_). Virus stock was stored at −80 °C, and the experimental inoculum was prepared from stock by dilution (10^–4^) with PBS. Shrimp were challenged by intramuscular injection with WSSV inoculum (100 µl/shrimp). This challenge titer resulted in a WSSV-induced cumulative mortality of 50% at 72 hpi.

### Staining of lipid droplets (LDs) in primary WSSV-infected shrimp hemocytes

At 12 and 24 h post virus injection, the hemolymph of 3 ~ 6 shrimps from the WSSV-injected and PBS-injected group was collected individually with an equal volume of anticoagulant solution (450 mM NaCl, 10 mM KCl, 10 mM EDTA, 10 mM Tris-HCl, pH 7.5). Each sample of hemocytes was then seeded onto a cover glass in duplicate with 2× Leibovitz’s 15 medium (Invitrogen; with 10% FBS, 1% glucose, 0.005% NaCl, 100 U/ml penicillin, 100 μg/ml streptomycin, 1.25 g/ml fungizone). After incubating for 20 min at room temperature, the hemocytes were washed three times with PBS and then incubated with BODIPY 493/503 (Invitrogen) at a working concentration of 0.1 mg/mL for 1 h in the dark at room temperature to stain the neutral lipid droplets. The hemocytes were then washed 3 more times with PBS before being fixed with 4% formaldehyde on ice. Next, the cells were stained for 3 min with DAPI at a working concentration of 1.5 × 10^−5 ^µg/ml (4′-6-diamidino-2-phenylindole dihydrochloride; Vector Laboratories Inc.) to counterstain the nuclei. After washing another 3 times with PBS, the hemocytes were incubated with Evans blue (0.2 μg/ml) for 1 minute to counterstain the cytoplasm. Finally, the cells on the cover glasses were mounted on microscope slides and the fluorescence was investigated using a fluorescence microscope.

### Determination of LDs in primary WSSV-infected shrimp hemocytes

After the LDs in primary WSSV-infected shrimp hemocytes were stained as described above, several fields were randomly selected from each slide for image capture and subsequent analysis. For each shrimp, at least 100 cells were investigated. Several metrics were used in this study to assess the dynamics of lipid droplets in shrimp hemocytes: (i) Cells with LDs/total cells (%); (ii) number of LD puncta in cells with LDs; (iii) cells with cytoplasmic LDs/total cells with LDs (%); (iv) cells with nuclear LDs/total cells with LDs (%); (v) number of cytoplasmic LD puncta per cell with LDs; (vi) number of nuclear LD puncta per cell with LDs; (vii) maximum diameter of the cytoplasmic LDs; (viii) maximum diameter of the nuclear LDs; (ix) total cross-sectional area (µm^2^) of the LDs in the cytoplasm; (x) total cross-sectional area (µm^2^) of the LDs in the nucleus. The data were then subjected to the statistical analysis as described below.

### Measurement of lipase activity in shrimp hemocyte, stomach and hepatopancreas during WSSV infection

Pooled samples of shrimp hemocytes, stomach and hepatopancreas were collected at 12 and 24 h post WSSV injection (3 shrimp/pool and 4 pools/group) and the lipase activity was measured using a commercial kit (Lipase Activity Fluorometric Assay Kit III; BioVision Inc.). Samples were homogenized in either 100 µl (hemocytes) or 200 µl (stomach and hepatopancreas) of ice-cold Lipase Assay Buffer, and centrifuged to remove the cell debris. The resulting lysates (50 μl/well) were transferred to a 96-well plate and 50 μl of the reaction mixture (48 μl Assay Buffer and 2 μl Lipase substrate) was added into each well. After incubation at 37 °C for 30 min (T1), the absorbance of each sample was measured at Ex/Em = 529/600 nm to obtain the A1 value. After incubating for another 30 min (T2), the absorbance of each sample was measured again to obtain the A2 value. A methylresorufin standard curve was generated from a series of concentrations ranging from 0 to 100 pmol/well and used to convert the difference in absorbance (A2–A1) to the corresponding amount of methylresorufin (the *B* value). The lipase activity was calculated as follows: Lipase activity (mU/mg) = Δ*B*/ (Δ*T* × mg), where mg is the weight of the tissue sample in each reaction mixture. The resulting data were then subjected to the statistical analysis as described below.

### Quantification of FFAs in shrimp hemocyte, hemolymph, stomach, and hepatopancreas during WSSV infection

A Free Fatty Acid Quantification Colorimetric/Fluorometric Kit (BioVision Inc.) was used to detect the unbound long-chain fatty acids in each shrimp sample. For the hemocyte and hemolymph samples, shrimp hemolymph was collected and the hemocytes were separated out by centrifugation. Hemocyte, hemolymph, stomach and hepatopancreas samples were homogenized with 200 μl chloroform-Triton X-100 (1% Triton X-100 in pure chloroform), and after centrifugation (10 min at 13,000 × *g*), the organic phase (lower phase) of each sample was collected, air dried at 50 °C until no solution was left, and then vacuum dried for 30 min. Next, the pellet (the dried lipids) from each sample was dissolved in 200 μl of Fatty Acid Assay Buffer and 50 μl of the resulting solution was transferred to a well on a 96-well plate. ACS Reagent (2 μl) was added and the mixture was incubated at 37 °C for 30 min. Finally, 50 μl of reaction mixture (44 µl Fatty Acid Assay Buffer, 2 µl Fatty Acid Probe, 2 µl Enzyme Mix, and 2 µl Enhancer) was added to the well and the mixture was incubated in the dark for 30 more minutes at 37 °C. For calibration, palmitic acid samples and blanks were used according to the manufacturer’s instructions. The absorbance of each sample was measured at Ex/Em = 535/590 nm, and a palmitic acid standard curve was generated from a series of concentrations ranging from 0 to 10 nmol. This was used to convert sample readings to the corresponding amount of FFA (*F*_a_) in each well. The FFA concentration was then calculated as follows: Fatty Acid Concentration (nmol/mg) = *F*_a_/*S*_mg_ where *S*_mg_ is the tissue weight of the sample in each reaction mixture. The resulting data were subjected to the statistical analysis as described below.

### Preparation of the Carnitine acyltransferase I (CPT1) inhibitor Etomoxir and FAS inhibitor C75

Stock solutions of CPT1 inhibitor Etomoxir (2[6(4-chlorophenoxy)hexyl]oxirane-2-carboxylate; Tocris Bioscience) and FAS inhibitor C75 (4-Methylene-2-octyl-5-oxotetrahydrofuran-3-carboxylic acid; ENZO Life Sciences) were prepared by dissolving in ddH_2_O and DMSO respectively. Before use, both stocks were diluted with PBS to the required concentration.

### Determination of ATP/ADP ratio in shrimp hemocytes

An ApoSENSOR ADP/ATP ratio assay kit (BioVision) was used to determine the ATP/ADP ratio in pooled hemocytes (4–5 pools; 4 shrimp per pool) collected at 12 and 24 hpi. The procedure was slightly modified from Li et al.^40^ and Chen et al.^41^. After hemolymph was collected from the challenged shrimp, it was pooled and centrifuged at 3000 × *g* for 10 min at 4 °C, and the resulting pellet of hemocytes was suspended in 50 μl nucleotide releasing buffer. This suspension was then added to wells containing 10 μl ATP monitoring enzyme and 90 μl Nucleotide Releasing Buffer on a 96-well ELISA plate. Background luminescence control was prepared with only ATP monitoring enzyme and nucleotide releasing buffer. To measure ATP level in hemocytes, the first measurement was made for the control (Data A) and the sample (Data B) after 2 min of incubation. To measure ADP level in hemocytes, a second measurement was made again for the sample (Data C). ADP converting enzyme (1 μl) was then added to the sample and a final measurement (Data D) was taken. All measurements were made using a luminometer. The ATP/ADP ratio was calculated as: (Data B) − (Data A)/[(Data D − Data A) − (Data C − Data A)].

### Quantification of WSSV genome copy numbers in shrimp hemocytes and hemolymph

To investigate the virion release, hemocytes were separated from hemolymph by centrifugation. The total genomic DNA was extracted from both hemocytes and hemolymph by using a DTAB/CTAB DNA extraction kit (GeneReach Biotechnology Corp.). The WSSV genome copy numbers were quantified by an IQ Real^TM^ WSSV quantitative system (GeneReach Biotechnology Corp.), a commercial real-time PCR, according to the manufacturer’s instructions.

### Using gas chromatography mass spectrometry (GC/MS) to analyze the effect of the CPT1 inhibitor Etomoxir and FAS inhibitor C75 on the LCFA profiles of stomach and hepatopancreas collected from WSSV-infected shrimp

To examine the role of β-oxidation and the LCFAs in WSSV replication, we used the CPT1 inhibitor Etomoxir and FAS inhibitor C75, respectively. For CPT1 inhibition, shrimp were intramuscularly injected with Etomoxir (62.5 mg/g shrimp) or PBS vehicle (control) 10 h after WSSV challenge. Pooled samples of hepatopancreas (4–5 pooled samples; 3 shrimp per pool) were collected at 12 and 24 hpi. GC/MS-based fatty acid profiling was used to monitor the dynamics of each detected long-chain fatty acid. For FAS inhibition, dosages and timing of C75 treatment were modified from our previous study^23^ Shrimp were treated with the FAS inhibitor C75 (30 μg/g shrimp) 4 h after being injected with WSSV or PBS. Hepatopancreas and stomach samples (4–5 pooled samples; 3 shrimp per pool) were collected at 12 and 24 hpi and used for the GC/MS analysis.

Gas chromatography mass spectrometry (GC/MS)-based shrimp lipodomics was performed based on the methods described by Hsieh et al.^23^. In short, a chloroform solution containing a C22:0 fatty acid internal control was first added to the shrimp tissues (10–30 mg per sample), and fatty acids were then extracted from the tissues by homogenization with 2:1 (v/v) chloroform/methanol. After overnight shaking and debris removal by filtration, the liquid fraction was dried at 40 °C using a rotary evaporator. The resulting pellets from each sample were then re-dissolved with pure chloroform, transferred into a 10 ml reaction vial and then dried again using the rotary evaporator. The dried samples were subjected to saponification/esterification followed by GC/MS lipodomics analysis using a 7890A gas chromatography machine equipped with a 5975C single-quadrupole mass spectrometer (Agilent, Palo Alto, CA, USA) and a Supelco SP-2380 capillary column (30 m x 0.25 mm i.d.; Sigma Aldrich, St. Louis, MO, USA) as described previously^23^. MSD Chemstation G1701EA E.02.02.1431 (Agilent Technologies, Inc., USA) was used for instrument control, data acquisition and data analysis. NIST 11 Mass Spectral Library (Gaithersburg, MD, USA) was used to assist the identification of FA methyl esters (FAMEs), along with the retention times of the standards purchased from Sigma-Aldrich. The identified fatty acids were quantified against the C22:0 fatty acid internal standard, and the amount of FA per mg of tissue was calculated. The resulting data were subjected to the statistical analysis as described below.

### Statistics and reproducibility

Data calculations and graphs were conducted in Microsoft Excel and Graphpad Prism 9 respectively. Raw data were collected from at least three independent replicates in each experiment, as described in detail in the corresponding method section. After the Empirical Rule was performed as previously described^51^ on all data for the detection and exclusion of statistical outliers, statistically significant differences between groups were analyzed either by Student’s *t* test for comparing multiple treatments. Data provided in Supplementary Data 1 are used to generate figures presented in this study. Data are presented as mean ± SD. Significance is indicated by *p < 0.05, **p < 0.01.

### Reporting summary

Further information on research design is available in the Nature Portfolio Reporting Summary linked to this article.

## Supplementary information

Wang_Peer Review File

Supplementary information

Description of Additional Supplementary Files

Supplementary Data 1

Reporting summary

## Data Availability

Source data for main figures presented in this study are provided as Supplementary Data 1. All other data are available from the corresponding author on reasonable request.
