# Supplementary material for: White spot syndrome virus (WSSV) modulates lipid metabolism in white shrimp
Source: Commun Biol. 2023 May 20;6:546. doi: 10.1038/s42003-023-04924-w (PMC10199447; doi:10.1038/s42003-023-04924-w)
Supplement: Supplementary file 2 — Supplementary information [file 42003_2023_4924_MOESM2_ESM.pdf]

Supplementary information

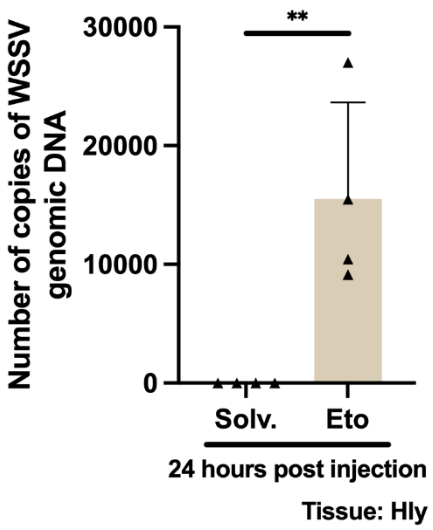

Figure S1. Inhibiting β-oxidation leads to an increase in WSSV genome copy number in the hemolymph of infected shrimp. Shrimp were injected with Etomoxir 10 hours after WSSV challenge. PBS served as the solvent control group. Hemolymph from 24 hpi was used to quantify the viral genome copy numbers to assess the virion count. Each bar represents mean ± SD, n = 4 pool samples (4 shrimp per pool). Asterisks indicate differences between WSSV and PBS groups (* $p<0.05$, ** $p<0.01$). Solv.: Solvent; Eto: Etomoxir; Hly: Hemolymph

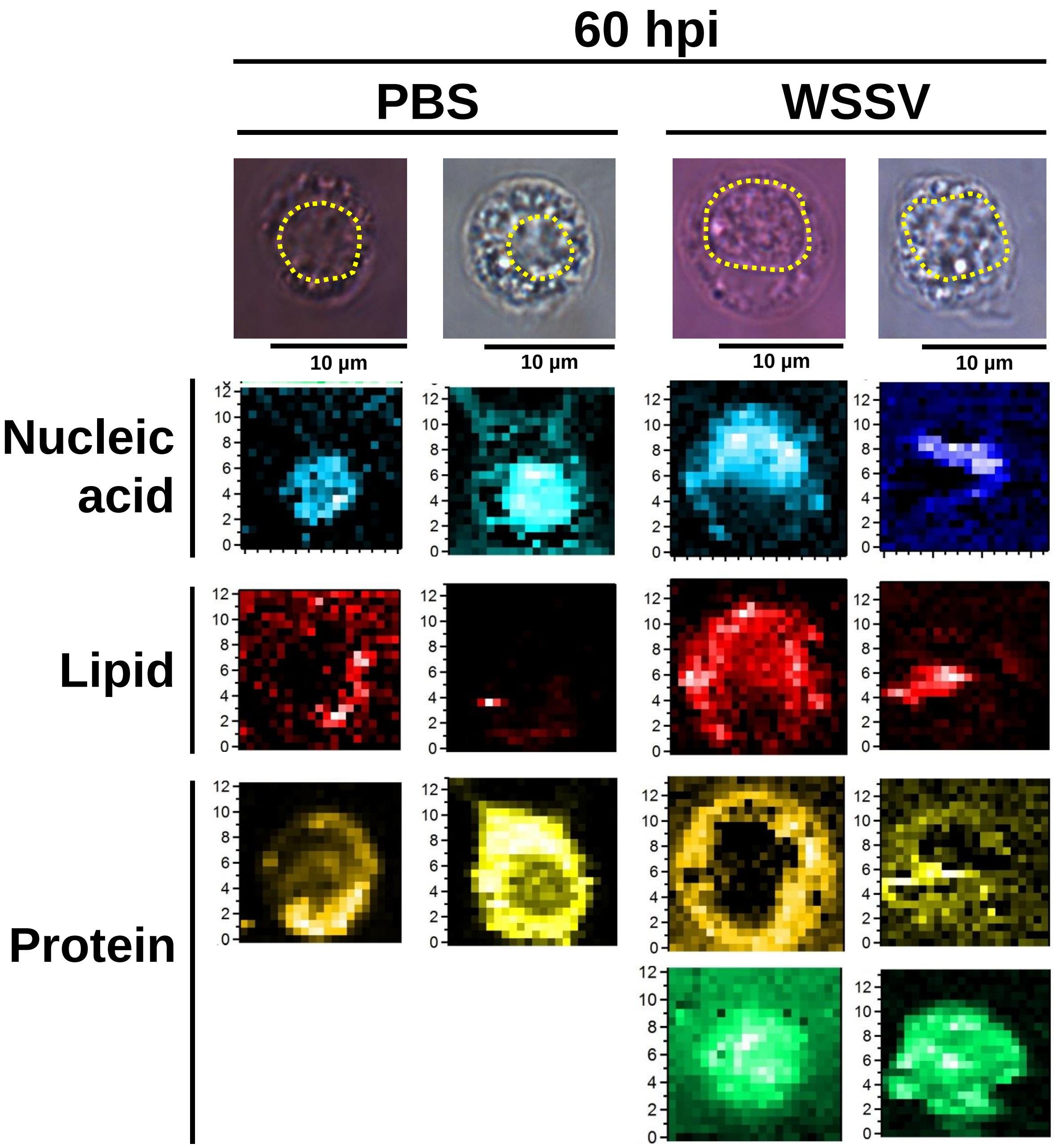

Figure S2. Multimode Raman imaging of WSSV-infected hemocytes. Kuruma shrimp (*Marsupenaeus japonicus*, 3g) were used in this experiment. WSSV-infected and control (PBS) hemocytes were collected at 60 hpi and subjected to Raman spectroscopy with 632.8 nm excitation. The Raman spectra was then analyzed by multivariate curve resolution (MCR) for visualization of biomolecules in cells. The detail specification and MCR analysis were previously described as Lai *et al.* (2015) [52] and Ando *et al.* (2014) [53]. The nucleus of hemocytes was labeled with yellow dotted line. The scale bar represents 10 µm.

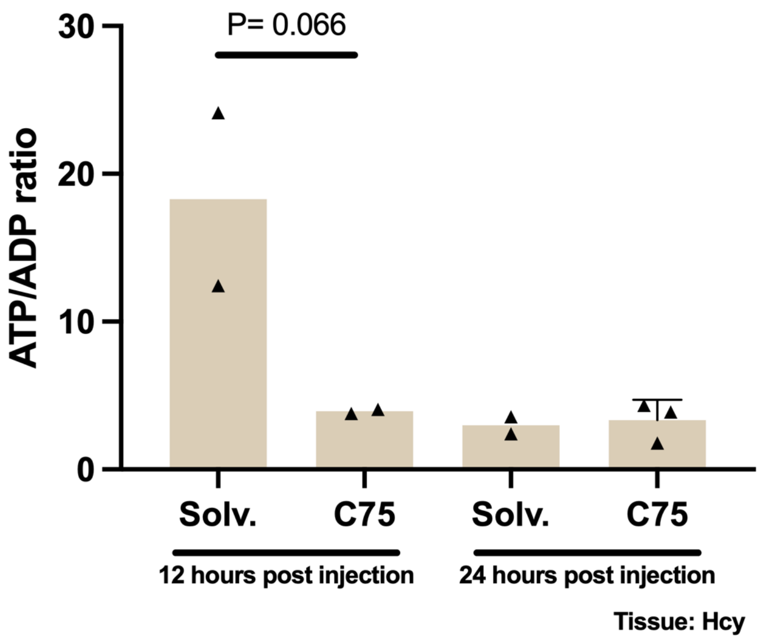

Figure S3. FAS inhibition did not change the energy state of infected hemocytes. The shrimp were injected with C75 4 hours after WSSV challenge. The hemocytes were collected at 12 and 24 hpi and subjected to ATP/ADP ratio quantification using an ApoSENSOR ADP/ATP ratio assay kit (BioVision, Inc.). Each bar represents mean ± SD, n = 2~3 pool samples (3 shrimp per pool). Solv.: Solvent; Hcy: Hemocytes.
