# Supplementary material for: White spot syndrome virus (WSSV) modulates lipid metabolism in white shrimp
Source: Commun Biol. 2023 May 20;6:546. doi: 10.1038/s42003-023-04924-w (PMC10199447; doi:10.1038/s42003-023-04924-w)
Supplement: Supplementary file 5 — Reporting summary [file 42003_2023_4924_MOESM5_ESM.pdf]

nature portfolio

Corresponding author(s): Han-Ching Wang

Last updated by author(s): Apr 22, 2023

Reporting Summary

Nature Portfolio wishes to improve the reproducibility of the work that we publish. This form provides structure for consistency and transparency in reporting. For further information on Nature Portfolio policies, see our Editorial Policies and the Editorial Policy Checklist.

Statistics

For all statistical analyses, confirm that the following items are present in the figure legend, table legend, main text, or Methods section.

n/a	Confirmed	
☐	☒	The exact sample size (n) for each experimental group/condition, given as a discrete number and unit of measurement
☐	☒	A statement on whether measurements were taken from distinct samples or whether the same sample was measured repeatedly
☐	☒	The statistical test(s) used AND whether they are one- or two-sided<br>*Only common tests should be described solely by name; describe more complex techniques in the Methods section.*
☒	☐	A description of all covariates tested
☒	☐	A description of any assumptions or corrections, such as tests of normality and adjustment for multiple comparisons
☐	☒	A full description of the statistical parameters including central tendency (e.g. means) or other basic estimates (e.g. regression coefficient) AND variation (e.g. standard deviation) or associated estimates of uncertainty (e.g. confidence intervals)
☐	☒	For null hypothesis testing, the test statistic (e.g. F, t, r) with confidence intervals, effect sizes, degrees of freedom and P value noted<br>*Give P values as exact values whenever suitable.*
☒	☐	For Bayesian analysis, information on the choice of priors and Markov chain Monte Carlo settings
☒	☐	For hierarchical and complex designs, identification of the appropriate level for tests and full reporting of outcomes
☒	☐	Estimates of effect sizes (e.g. Cohen's d, Pearson's r), indicating how they were calculated

Our web collection on statistics for biologists contains articles on many of the points above.

Software and code

Policy information about availability of computer code

Data collection	For GC/MS, MSD Chemstation G1701EA E.02.02.1431 (Agilent Technologies, Inc., USA) was used for instrument control, data acquisition and data analysis. NIST 11 Mass Spectral Library (Gaithersburg, MD, USA) was used to assist the identification of FA methyl esters (FAMEs), along with the retention times of the standards purchased from Sigma-Aldrich.
Data analysis	Microsoft excel was used for data calculation. Graphpad Prism 9 was used for diagram visualization.

For manuscripts utilizing custom algorithms or software that are central to the research but not yet described in published literature, software must be made available to editors and reviewers. We strongly encourage code deposition in a community repository (e.g. GitHub). See the Nature Portfolio guidelines for submitting code & software for further information.

Data

Policy information about availability of data

All manuscripts must include a data availability statement. This statement should provide the following information, where applicable:

- Accession codes, unique identifiers, or web links for publicly available datasets
- A description of any restrictions on data availability
- For clinical datasets or third party data, please ensure that the statement adheres to our policy

All data are available from the corresponding author on reasonable request.

Human research participants

Policy information about studies involving human research participants and Sex and Gender in Research.

Reporting on sex and gender	n/a
Population characteristics	n/a
Recruitment	n/a
Ethics oversight	n/a

Note that full information on the approval of the study protocol must also be provided in the manuscript.

Field-specific reporting

Please select the one below that is the best fit for your research. If you are not sure, read the appropriate sections before making your selection.

☒ Life sciences ☐ Behavioural & social sciences ☐ Ecological, evolutionary & environmental sciences

For a reference copy of the document with all sections, see nature.com/documents/nr-reporting-summary-flat.pdf

Life sciences study design

All studies must disclose on these points even when the disclosure is negative.

Sample size	The sample size was based on preliminary experiments and publications by us and other groups.
Data exclusions	The Empirical Rule was performed on all data for the detection and exclusion of statistical outliers, statistically significant differences between groups were analyzed either by Student's t-test.
Replication	Replicates for each experiment are indicated in the method. Each experiment was performed with at least three replicates.<br>Biological replicates are presented as mean with standard deviation.
Randomization	shrimp were distributed at random over experimental groups.
Blinding	n/a

Reporting for specific materials, systems and methods

We require information from authors about some types of materials, experimental systems and methods used in many studies. Here, indicate whether each material, system or method listed is relevant to your study. If you are not sure if a list item applies to your research, read the appropriate section before selecting a response.

Materials & experimental systems

n/a	Involved in the study	
☒	☐	Antibodies
☒	☐	Eukaryotic cell lines
☒	☐	Palaeontology and archaeology
☐	☒	Animals and other organisms
☒	☐	Clinical data
☒	☐	Dual use research of concern

Methods

n/a	Involved in the study	
☒	☐	ChIP-seq
☒	☐	Flow cytometry
☒	☐	MRI-based neuroimaging

Animals and other research organisms

Policy information about studies involving animals; ARRIVE guidelines recommended for reporting animal research, and Sex and Gender in Research

Laboratory animals	Specific pathogen free white shrimp (Litopenaeus vannamei; 3g body weight) were used in this study and obtained from Aquatic Animal Center at National Taiwan Ocean University, the International Center for the Scientific Development of Shrimp Aquaculture,

	National Cheng Kung University (NCKU) and the Department of Aquaculture, National Pingtung University of Science and Technology (NPUST).
Wild animals	This study did not involve wild animals.
Reporting on sex	This study focuses on virus-modulated lipogenesis in shrimp, therefore, sex-based analysis is not relevant to our study.
Field-collected samples	This study did not involve samples collected from field.
Ethics oversight	The experimental animals (shrimp) were invertebrates, which no specific permits were required for this study.

Note that full information on the approval of the study protocol must also be provided in the manuscript.
