# Supplementary material for: White spot syndrome virus (WSSV) modulates lipid metabolism in white shrimp
Source: Commun Biol. 2023 May 20;6:546. doi: 10.1038/s42003-023-04924-w (PMC10199447; doi:10.1038/s42003-023-04924-w)
Supplement: Supplementary file 1 — Wang_Peer Review File [file 42003_2023_4924_MOESM1_ESM.pdf]

nature portfolio

Peer Review File

White spot syndrome virus (WSSV) modulates lipid metabolism in white shrimp

Open Access This file is licensed under a Creative Commons Attribution 4.0 International License, which permits use, sharing, adaptation, distribution and reproduction in any medium or format, as long as you give appropriate credit to the original author(s) and the source, provide a link to the Creative Commons license, and indicate if changes were made. In the cases where the authors are anonymous, such as is the case for the reports of anonymous peer reviewers, author attribution should be to 'Anonymous Referee' followed by a clear attribution to the source work. The images or other third party material in this file are included in the article's Creative Commons license, unless indicated otherwise in a credit line to the material. If material is not included in the article's Creative Commons license and your intended use is not permitted by statutory regulation or exceeds the permitted use, you will need to obtain permission directly from the copyright holder. To view a copy of this license, visit http://creativecommons.org/licenses/by/4.0/.

Reviewers' comments:

Reviewer #1 (Remarks to the Author):

In this manuscript, the authors reported that WSSV caused a reduction in LDs in hemocytes at the viral genome replication stage, and an increase in LDs in the nuclei of WSSV-infected hemocytes at the viral late stage. In the hepatopancreas, lipolysis was triggered by WSSV infection, and this led to fatty acids being released into the hemolymph. Aseries of β-oxidation inhibition experiments revealed that the fatty acids generated by WSSV-induced lipolysis can be diverted either into β-oxidation for energy production or else used as material for virion morphogenesis. At the viral late stage, WSSV infection led to lipogenesis in both the stomach and hepatopancreas, suggesting that fatty acids are in high demand at this stage for virion morphogenesis. On the whole, the authors investigated the mechanisms behind these lipid metabolism shifts in WSSV-infected shrimp. The amount of work is substantial and seems to be of good quality. However, there are still some questions and suggestions regarding the experimental design and article presentation.

Some comments:
1. In Figure 1B (i), Cells with LDs decreased at 12h, while in (iii) and (iv), neither the cytoplasm nor the nucleus decreased significantly. Is it reasonable?

2. Figure 1A (vii-x), What is the significance of LD diameter and area measurements? Why was there a significant decrease in both LD diameter and area at 24h?

3. Figure 2, the authors examined the lipase activity in three different tissues of WSSV-infected shrimp. At the viral genome replication stage, lipase activity was elevated in the WSSV-infected hepatopancreas and WSSV-infected hemocytes at the late stage, However, there was no corresponding upregulation of the corresponding free fatty acids in Figure2C, What explains this phenomenon?

4. Line281-284, “FFAs were significantly decreased in WSSV-infected hepatopancreas at the viral late stage (Fig. 2C), which might be a consequence of lipid depletion during the viral genome replication stage”. Why is FFA not reduced by viral utilization in other cell types?

5. Line287-289, “Under normal circumstances, lipid droplets and triglycerides are the substrates of lipase, and they can be degraded into free fatty acids which are then transported into the mitochondria and used to produce energy by β-oxidation”. , However, in Figure 2A, the ATP/ADP level was increased when the β-oxidation was inhibited, is the result reasonable?

6. Line309-311, “To account for this, we hypothesized that the LCFAs produced by the lipase activity might be shifted to β-oxidation, while noting that this would also be consistent with the increased virion morphogenesis seen in Figure 3B”.As shown in Figure3, β-oxidation appears to inhibit WSSV replication. What is the logic of this sentence, please reformulate it?

8.The detection method in this article is relatively single, especially the detection part of lipid droplets, and some effects are not obvious enough to explain the conclusion.

Reviewer #2 (Remarks to the Author):

What are the main claims of the article?
1- The authors have found evidence of the regulation of fluid metabolism in the host during WSSV infection.
2- Lipolysis and lipogenesis are controlled in the early phase of

and in the late phase, respectively, during WSSV infection.
Are they novel and will they be of interest to others in the community and the wider field?
1- They postulate that this new knowledge can help to understand the evolution of the infection.
2- They have shed light on how the virus diverts lipid metabolism to obtain energy during the early stages of infection and finally on the need to generate specific lipids that will allow it to achieve the lipid envelope of the mature virion.
Is the work convincing and, if not, what additional evidence would be required to strengthen the conclusions?
The analytical techniques used by the authors are adequate and reliable for obtaining the results they describe, of which they have proposed their discussion compared with other families of viruses such as the flaviviridea that hijack the metabolism of lipids in arthropods such as the mosquito, Similarly.
On a more subjective note, do you feel the paper will influence thinking in the field?
The authors suggest that such results can help to better understand the distribution of fatty acids in such conditions, but that more studies are needed, such as the use of isotopes to trace their course and define whether essential lipids such as cholesterol are involved in the process.

Reviewer #3 (Remarks to the Author):

The manuscript provide a broad picture on lipid metabolism during WSSV infection in shrimp using a sound experimental approach with several linked methodologies. The manuscript is well written, supported and discussed leading to an integrated conclusion, although some details could be improved as suggested below.
Material and Methods
There is an excessive use of "The" at the beginning of each sentence
Line 102 and 103 I think this sentence is useless, you can just mention the GenBank reference on line 106 after Taiwan isolate
Line 113 Time elapsed from infection and hemolymph collection should be mentioned here (12 and 24 hpi)
ATP/ADP methodology is confusing, and no reference is provided. I think that more details should be provided to clearly understand the meaning of data A, B, C and D

Results
Several portions of the results section seem to be more suitable in other sections. For example, sentences on lines 266-267; 281-282; 283-284; 301-303; 335-337, 342-345 provide an interpretation of results and therefore should be in the Discussion section. Alternatively, Results and Discussion should be fused if the journal allows a combined Results and Discussion section.
Besides, other sentences explain why a particular experimental approach was used. This is the case for the beta oxidation inhibition and the subsequent determination of ATP/ADP ratio (lines 287-292), viral replication (lines 295-296) and fatty acid content (lines 307 and 311), as well as for fatty acid synthesis (lines 325-330). I think that these sentences are better suited in the Material and Methods section as in a first instance, I did not understand why beta oxidation was inhibited when reading this section.

Discussion
Results of ATP/ADP ratio are nor discussed. If we look only at data with solvent only, ATP/ADP ratio increased; a similar result of increased adenylic energy charge in hepatopancreas of shrimp following infection with WSSV (Apun-Molina et al., Aquaculture 479: 528-3537, 2017 http://dx.doi.org/10.1016/j.aquaculture.2017.06.027. In contrast Che et al 2011 (already cited in the MS) observed a decrease in ATP at later (over 24 hpi) stages of infection and proposed a dual time effect, similarly to the present study although based on carbohydrate metabolism. The results with Eto also should be discussed as the contribution of Beta-oxidation to the increase in ATP production (ATP/ADP ratio) during the early phase of infection which is in accordance with the general conclusion of the present study. However other results remain to be explained; for example, why such increase in

ATP production is sustained at later stages of infection and apparently is independent of beta oxidation.

Responses to the reviewers' comments:

Reviewer #1 .

Comment 1:
In Figure 1B (i), Cells with LDs decreased at 12h, while in (iii) and (iv), neither the cytoplasm nor the nucleus decreased significantly. Is it reasonable?
Response: Thank you for catching this. Figure 1b(i) was to understand the percentage of LD-detected cells in all of the investigated cells. However, for Figure 1b(iii) and (iv), we compared the percentages of LDs in the LD-detected cell populations only. This was correctly described in methods (lines 308-309) but was inadvertently omitted from the Figure legend. The Figure 1 legend has now been revised to include this information (lines 587-588).

Comment 2:
Figure 1A (vii-x), What is the significance of LD diameter and area measurements? Why was there a significant decrease in both LD diameter and area at 24h?
Response: We too are interested in understanding the fluctuations of size of LDs during the WSSV infection, and hope it might provide an insight into WSSV-modulated lipid metabolism. However, in this case, although the nucleus LDs was not increased in size at 24 hpi, we can only speculate that the size decrease of cytoplasm LDs might be due to the redistribution of lipid to the nucleus, which facilitates the virion assembly at the late stage.

Comment 3:
Figure 2, the authors examined the lipase activity in three different tissues of WSSV-infected shrimp. At the viral genome replication stage, lipase activity was elevated in the WSSV-infected hepatopancreas and WSSV-infected hemocytes at the late stage, However, there was no corresponding upregulation of the corresponding free fatty acids in Figure2C, What explains this phenomenon?
Response: The hepatopancreas is thought to be a lipid-storage organ in shrimp. When lipolysis occurred in the WSSV-infected hepatopancreas at 12 hpi, free fatty acids (FFAs) were released into hemolymph and taken up by hemocytes (Fig 2c). This might explain why there was no upregulation of FFAs in the infected hepatopancreas. We discuss this phenomenon in line 233 to line 237. (This passage has been slightly revised from the originally submitted version.)
"Figure 2a shows that lipase activity was triggered in shrimp hepatopancreas, which is a lipid-storing tissue. Unlike the total fatty acids, which Hsieh et al. (2015) found to be decreased in the stomach at 12 hpi and increased at 24 hpi, what we found here suggests that at 12 hpi, free fatty acids (FFAs) might have been released from the hepatopancreas into the hemolymph and then taken up by hemocytes (Fig. 2c)."

Comment 4:
Line281-284, "FFAs were significantly decreased in WSSV-infected hepatopancreas at the viral late stage (Fig. 2C), which might be a consequence of lipid depletion during the viral genome replication stage". Why is FFA not reduced by viral utilization in other cell types?
Response: This is a deeply interesting question! Although WSSV invades every tissue of shrimp with different degree of infectivity, we hypothesize that the virus might specifically target the

hepatopancreas through unknown mechanisms, and that this in turn activates a higher activity of lipase relative to the lipase activity in other infected tissues. This can ensure the sufficient supply of fatty acid for the virus replication in other tissues. We note that selective infection is also found in HIV where it selectively infects effector memory T cells, which have higher glycolysis and oxidative phosphorylation than other T cells subsets (Valle-Casuso et al., 2018). However, more work will need to be done to understand whether there is any hepatopancreas-specific mechanism that can employed by WSSV to elevate the lipase activity.

Reference
Valle-Casuso, J. C., Angin, M., Volant, S., Passaes, C., Monceaux, V., Mikhailova, A., Bourdic, K., Avettand-Fenoel, V., Boufassa, F., Sitbon, M., Lambotte, O., Thoulouze, M. I., Müller-Trutwin, M., Chomont, N., & Sáez-Cirión, A. Cellular Metabolism Is a Major Determinant of HIV-1 Reservoir Seeding in $CD4^+$ T Cells and Offers an Opportunity to Tackle Infection. *Cell Metab.* 29, 611-626.e5. (2019)

Comment 5:

Line287-289, "Under normal circumstances, lipid droplets and triglycerides are the substrates of lipase, and they can be degraded into free fatty acids which are then transported into the mitochondria and used to produce energy by β-oxidation". , However, in Figure 2A, the ATP/ADP level was increased when the β-oxidation was inhibited, is the result reasonable?

Response: It is interesting to observe the ATP/ADP level was increased after β-oxidation inhibition in infected and non-infected group at 24 hpi (Figure 3a, Right panel). We speculate that other energy pathways might be activated after the long duration of β-oxidation inhibition in order to maintain the viability of the cell. This has now been added to the discussion about ATP/ADP levels at 24 hpi in line 227 to line 232"

"As discussed earlier, β-oxidation might be providing ATP for virus replication at the genome replication stage. Surprisingly however, at 24 hpi, β-oxidation inhibition resulted in elevated ATP levels regardless of infection status (Figure 3a, Solvent group vs Etomoxir group at 24 hpi). We speculate that the long duration of β-oxidation inhibition might have led to the activation of other energy pathways in order to maintain cell viability."

Comment 6:

Line309-311, "To account for this, we hypothesized that the LCFAs produced by the lipase activity might be shifted to β-oxidation, while noting that this would also be consistent with the increased virion morphogenesis seen in Figure 3B".As shown in Figure3, β-oxidation appears to inhibit WSSV replication. What is the logic of this sentence, please reformulate it?

Response: We agree that there is a problem with our argument here. This passage has been re-written as follows (line 143 to line 146).

"Lipase was activated in the hepatopancreas at the viral genome replication stage (Fig. 2a), and we hypothesized that the LCFAs produced by the lipase activity might be shifted to β-oxidation. This would also be consistent with Hsieh *et al*. (2015), who reported that various LCFAs were decreased in infected stomach at the same stage."

Comment 8:
The detection method in this article is relatively single, especially the detection part of lipid droplets, and some effects are not obvious enough to explain the conclusion.
Response: We appreciate that there were limitations to our means of detection. However, as described in methods (lines 292-300), the lipid droplets (LDs) in the hemocytes were stained using BODIPY 493/503 (Invitrogen). And with the staining of cytoplasm and nucleus, we were able to distinguish whether the LDs were from the cytoplasm or nucleus.

Reviewer #2 .

Comment 1.
1-**The authors have found evidence of the regulation of fluid metabolism in the host during WSSV infection.**
2-Lipolysis and lipogenesis are controlled in the early phase of and in the late phase, respectively, during WSSV infection.
Are they novel and will they be of interest to others in the community and the wider field?
Response: Our study demonstrated that WSSV induces lipolysis and lipogenesis in viral genome replication stage (12 hpi) and viral late stage (24 hpi), respectively. This dual regulation of lipid metabolism is also reported in flaviviruses, a single-stranded RNA virus. To our knowledge, our study is the first to report that double-stranded DNA virus like WSSV also could trigger a similar lipid regulation as flaviviruses. Additionally, we demonstrated the timepoint for lipolysis and lipogenesis occurrence during the first virus replication cycle, which has not previously been reported in other virus research. We believe our study supports the idea that viruses, regardless of DNA virus or RNA virus, modulate the lipid metabolism specifically at different stages of infection in order to obtain necessities at that timepoint for their replication.

Comment 2.
1- They postulate that this new knowledge can help to understand the evolution of the infection.
2- They have shed light on how the virus diverts lipid metabolism to obtain energy during the early stages of infection and finally on the need to generate specific lipids that will allow it to achieve the lipid envelope of the mature virion.
Is the work convincing and, if not, what additional evidence would be required to strengthen the conclusions?
Response: We are confident that our results sufficiently support our conclusions, which provide an overview of the lipid metabolism during WSSV replication. However, several questions remained to be answered.

A. Which fatty acid is specifically required by WSSV for virion assembly and where are they generated from? Zhou et al. (2008) reported that certain hydrocarbon and cholesterol species are enriched in the WSSV virion. We hypothesize that the generation of these WSSV-required lipid species may occur using the material from lipolysis at viral genome replication stage (12 hpi). Techniques like isotope tracing would help to further understand the route of lipid metabolism over the WSSV replication. We already established an *in vivo* isotope tracing system in He et al. (2019) and Ng et al. (2022) and we are currently working on answering this question.

B. What is the role of the accumulated nucleus LDs in WSSV replication? We observed that LDs were accumulated in the nucleus of the cells from the viral late stage, but we still don't know how this is involved in virion assembly. Two hypotheses have been made to explain this phenomenon. (1) the accumulated LDs might regulate gene expression that facilitates viral morphogenesis. (2) the accumulated LDs serves as a viral protein-storage depot for virus assembly. To address these questions, Transcriptomics analysis combined with LD formation inhibition might give us an insight into which gene expressions are affected when the LDs can't be accumulated. Identification of LD proteins that interact with viral proteins might also help us to understand the role of LDs during virus replication.

References:

1. Zhou, Q., Li, H., Qi, Y., & Yang, F. Lipid of white-spot syndrome virus originating from host-cell nuclei. *J. Gen. Virol.* 89, 2909-2914. (2008)
2. He, S., Lee, D., Tung, C., Li, C., & Wang, H. Glutamine metabolism in both the oxidative and reductive directions is triggered in shrimp immune cells (hemocytes) at the WSSV genome replication stage to benefit virus replication. *Front. Immunol.* 10, 2102. (2019)
3. Ng, Y. S., Lee, D. Y., Liu, C. H., Tung, C. Y., He, S. T., & Wang, H. C. White Spot Syndrome Virus Triggers a Glycolytic Pathway in Shrimp Immune Cells (Hemocytes) to Benefit Its Replication. *Front Immunol.* 13, 901111. (2022)

Comment 3:

The analytical techniques used by the authors are adequate and reliable for obtaining the results they describe, of which they have proposed their discussion compared with other families of viruses such as the flaviviridea that hijack the metabolism of lipids in arthropods such as the mosquito, Similarly. On a more subjective note, do you feel the paper will influence thinking in the field?

The authors suggest that such results can help to better understand the distribution of fatty acids in such conditions, but that more studies are needed, such as the use of isotopes to trace their course and define whether essential lipids such as cholesterol are involved in the process.

Response: Thank you for the feedback. We hope our study can prompt the community to focus on the changes of lipid metabolism over the virus replication process. Although virus-regulated metabolism was reported in early years of virus research, investigation of lipid metabolism is still relatively new, especially in WSSV. Most virus research has focused on the lipid metabolism from a certain timepoint or stage of virus infection, which means that the changes in lipid metabolism during the course of virus replication remain largely unknown. We believe that viruses, as evolving parasites, are able to adjust their host's lipid metabolism to provide necessities at specific timepoints. This can be done through the breakdown (lipolysis) or generation (lipogenesis) of the lipid. The lipid breakdown and generation also can be seen in Dengue virus (DENV) infection to facilitate their replication (Tang et al., 2014; Zhang et al., 2018). Additionally, Samsa et al. (2009) reported that DENV capsid protein usurps LDs for viral particle formation. Examples from DENV infection show that virus regulates LDs to achieve certain goals during virus replication. Our study demonstrated a similar result, in which lipolysis at the early stage was to provide energy and, lipogenesis at the late stage to provide fatty acids for virus replication. Our study together with DENV examples might provide a research direction, where the role of lipid (in the form of LDs, fatty acids or others) in virus replication is investigated in more detail.

References

1. Tang, W. C., Lin, R. J., Liao, C. L., & Lin, Y. L. Rab18 facilitates dengue virus infection by targeting fatty acid synthase to sites of viral replication. *J. Virol.* 88, 6793–6804. (2014)
2. Zhang, J., Lan, Y., Li, M. Y., Lamers, M. M., Fusade-Boyer, M., Klemm, E., Thiele, C., Ashour, J., Sanyal, S., Flaviviruses Exploit the Lipid Droplet Protein AUP1 to Trigger Lipophagy and Drive Virus Production. *Cell Host Microbe*. 23, 819-831.e5. (2018)
3. Samsa, M. M., Mondotte, J. A., Iglesias, N. G., Assunc¸a˜o-Miranda, I., Barbosa-Lima, G., Poian, A. T. D., Bozza, P. T., & Gamarnik, A.V., Dengue virus capsid protein usurps lipid droplets for viral particle formation. *PLoS Pathog.* 5, e1000632. (2009)

Reviewer #3 .

Comment 1:
There is an excessive use of "The" at the beginning of each sentence
Response: Thank you for your advice, we have now revised our manuscript to minimize the usage of "The".

Comment 2:
Line 102 and 103 I think this sentence is useless, you can just mention the GenBank reference on line 106 after Taiwan isolate
Response: We removed the sentence from line 102 and 103 and added the GenBank reference in line 279 after Taiwan isolate.

Comment 3:
Line 113 Time elapsed from infection and hemolymph collection should be mentioned here (12 and 24 hpi)
Response: We have re-written the sentence (line 287 to line 289) to emphasize the timepoint of collection.
"At 12 and 24 hours post virus injection, the hemolymph of 3~6 shrimps from the WSSV-injected and PBS-injected groups was collected individually with an equal volume of anticoagulant solution (450 mM NaCl, 10 mM KCl, 10 mM EDTA, 10 mM Tris-HCl, pH 7.5)."

Comment 4:
ATP/ADP methodology is confusing, and no reference is provided. I think that more details should be provided to clearly understand the meaning of data A, B, C and D
Response: ATP/ADP determination has been reported in our previous study [Li et al. (2016) and Chen et al. (2011)]. We now included the reference in line 368. Besides, we apologized for the unclear data A, B, C and D. The ATP measurement was first conducted on the control and sample, which the result was named Data A and Data B respectively. To measure the ADP level, the measurement was read again for the sample, which is Data C. ADP converting enzyme was then added to the sample to convert the ADP to ATP. Lastly the final measurement was done again on the sample, which is Data D. ATP/ADP ratio was calculated as: (Data B) - (Data A) / [(Data D - Data A) - (Data C - Data A)]. We have slightly edited the method to describe the procedure more clearly (line 374 to line 379).
References

1. Chen, I. T., Aoki, T., Huang, Y. T., Hirono, I., Chen, T. C., Huang, J. Y., Chang, G. D., Lo, C. F., & Wang, H. C. White spot syndrome virus induces metabolic changes resembling the warburg effect in shrimp hemocytes in the early stage of infection. *J. Virol.* 5, 12919-12928. (2011)
2. Li, C. Y., Wang, Y. J., Huang, S. W., Cheng, C. S., & Wang, H.C. Replication of the Shrimp Virus WSSV Depends on Glutamate-Driven Anaplerosis. PLoS One. 11, e0146902. (2016)

Comment 5:
Results
Several portions of the results section seem to be more suitable in other sections. For example, sentences on lines 266-267; 281-282; 283-284; 301-303; 335-337, 342-345 provide an interpretation of results and therefore should be in the Discussion section. Alternatively, Results and Discussion should be fused if the journal allows a combined Results and Discussion section.
Besides, other sentences explain why a particular experimental approach was used. This is the case for the beta oxidation inhibition and the subsequent determination of ATP/ADP ratio (lines 287-292), viral replication (lines 295-296) and fatty acid content (lines 307 and 311), as well as for fatty acid synthesis (lines 325-330). I think that these sentences are better suited in the Material and Methods section as in a first instance, I did not understand why beta oxidation was inhibited when reading this section.
Response: Thank you for these comments. After discussed with other co-authors, we decided not to change our format. We think that using these brief introductory sentences in the results section can help the reader to understand the progression of the experiments in this study, which first showed that WSSV modulated LDs during infection, and then followed this up with further investigations into this phenomenon. We agree however that it would be helpful to introduce the methods of the β-oxidation experiments in the same way. In order to explain the reasons behind the β-oxidation experiments, we have therefore added "To examine the role of β-oxidation and the LCFAs in WSSV replication, we used the CPT1 inhibitor Etomoxir and FAS inhibitor C75, respectively." at line 392.

Comment 6:
Discussion
Results of ATP/ADP ratio are nor discussed. If we look only at data with solvent only, ATP/ADP ratio increased; a similar result of increased adenylic energy charge in hepatopancreas of shrimp following infection with WSSV (Apun-Molina et al., Aquaculture 479: 528-3537, 2017 http://dx.doi.org/10.1016/j.aquaculture.2017.06.027. In contrast Che et al 2011 (already cited in the MS) observed a decrease in ATP at later (over 24 hpi) stages of infection and proposed a dual time effect, similarly to the present study although based on carbohydrate metabolism. The results with Eto also should be discussed as the contribution of Beta-oxidation to the increase in ATP production (ATP/ADP ratio) during the early phase of infection which is in accordance with the general conclusion of the present study. However other results remain to be explained; for example, why such increase in ATP production is sustained at later stages of infection and apparently is independent of beta oxidation.

Response: We agree with this suggestion as a discussion about ATP/ADP ratio results could further explain our observations and provide a useful comparison with previous results. We have therefore added a new section discussing the ATP/ADP ratio results in line 221 to line 232.

Reviewers' comments:

Reviewer #1 (Remarks to the Author):

This manuscript mainly revealed the mechanisms behind lipid metabolism shifts in WSSV-infected shrimp. The authors reported the localization of lipid droplets in infected hemocytes at different stages of virus replication. And further results showed the activity of lipase, a key enzyme in lipolysis, and monitor its effect on the amount of fatty acids in shrimp tissue. Moreover, authors used Etomoxir, a CPT1 inhibitor, to block β-oxidation in WSSV-infected shrimp and investigate how β-oxidation affects energy production and virion morphogenesis. The above results show how WSSV is able to regulate the switch from lipolysis to lipogenesis at a specific time point after infection and establish a favorable environment for its replication. The amount of work is substantial and seems to be of good quality. Some questions and suggestions are to be noted.
Some comments:
1 The authors emphasized the number of lipid droplets in hemocytes was significantly increased in the WSSV infection group at the viral late stage (24 hpi). However, no obvious moreipid droplets was observed in hemocytes in the WSSV infection group.
2. Is there any decrease of total hemocytes number after WSSV challenge?
3. Fig 2b, at the late stage (24 hpi), WSSV enhances the expression of fatty acid synthase (FAS) to support lipogenesis. Why lipase activity was not changed in all WSSV-infected tissues?
4. Fig 3b, figure note errors?
5. An increase in LDs in the nuclei of WSSV-infected hemocytes at the viral late stage. Why the mitochondrial activity in WSSV-infected hemocytes was significantly increased at the late stage when the β-oxidation was inhibited?
6.The authors showed that blocking β-oxidation prevented further metabolic processing of the LCFAs induced by WSSV. Why the inhibition of β-oxidation consistently led to an increase in virion particles in both hemocytes and hemolymph?

Reviewer #3 (Remarks to the Author):

Authors have adequately revised their manuscript in line to previous comments

Responses to the reviewers' comments:

Reviewer #1 .

Comment 1:

The authors emphasized the number of lipid droplets in hemocytes was significantly increased in the WSSV infection group at the viral late stage (24 hpi). However, no obvious moreipid droplets was observed in hemocytes in the WSSV infection group.

Response: Result 1b(ii) demonstrated WSSV-infected hemocytes contained more lipid droplets compared to uninfected group. Additionally, more lipid droplets were also found in both cytoplasm and nuclear of the WSSV-infected hemocytes (Figure v and vi). The results have been discussed in result section (line 98 to 105).

Comment 2:

Is there any decrease of total hemocytes number after WSSV challenge?

Response: This is an interesting question. Although we did not conduct hemocyte count in this study, Tang et al. (2019) and Sahul Hammed et al. (2006) reported that hemocytes were decreased in a WSSV infection group compared to an uninfected group. This was mainly due to apoptosis induced by WSSV and associated with the release of virion progeny. In the Tang et al (2019) study, the largest increase in the number of apoptotic hemocytes (up to 20% of the total hemocyte population) occurred after the first infection cycle (ie after 24hpi), although there was also an increased number of apoptotic hemocytes at 12hpi. Meanwhile Cui et al (2020) found that the apoptotic rate of virus-negative hemocytes was significantly higher than that of virus-positive hemocytes in the early stage of WSSV infection. Clearly, this is a topic that calls for further study.

Reference

1. Cui, C., Liang, Q., Tang, X., Xing, J., Sheng, X., & Zhan, W. Differential Apoptotic Responses of Hemocyte Subpopulations to White Spot Syndrome Virus Infection in Fenneropenaeus chinensis. *Front immunol*, 11, 594390. (2020)
2. Sahul Hameed, A.S., Sarathi, M., Sudhakaran, R., Balasubramanian, G., & Syed Musthaq, S. Quantitative assessment of apoptotic hemocytes in white spot syndrome virus (WSSV)-infected penaeid shrimp, Penaeus monodon and Penaeus indicus, by flow cytometric analysis. *Aquaculture*, 256, 111-120. (2006)
3. Tang, X., Cui, C., Liang, Q., Sheng, X., Xing, J., & Zhan, W. Apoptosis of hemocytes is associated with the infection process of white spot syndrome virus in Litopenaeus vannamei. *Fish Shellfish Immunol.* 94, 907-915. (2019)

Comment 3:

Fig 2b, at the late stage (24 hpi), WSSV enhances the expression of fatty acid synthase (FAS) to support lipogenesis. Why lipase activity was not changed in all WSSV-infected tissues?

Response: It is interesting to observe that only hepatopancreas lipase activity was increased at 12hpi. We hypothesize that virus might specifically activate lipase in hepatopancreas since it is a lipid-storage organ. This can ensure the adequate supply of fatty acid for the virus replication in other tissues. We also note that HIV selectively infects effector memory T cells, and that these

have higher glycolysis and oxidative phosphorylation than other T cells subsets (Valle-Casuso et al., 2018). However, more study will need to be done to investigate whether there are any hepatopancreas-specific mechanisms that are involved in WSSV-induced lipase activity.

Reference
Valle-Casuso, J. C., Angin, M., Volant, S., Passaes, C., Monceaux, V., Mikhailova, A., Bourdic, K., Avettand-Fenoel, V., Boufassa, F., Sitbon, M., Lambotte, O., Thoulouze, M. I., Müller-Trutwin, M., Chomont, N., & Sáez-Cirión, A. Cellular Metabolism Is a Major Determinant of HIV-1 Reservoir Seeding in $CD4^+$ T Cells and Offers an Opportunity to Tackle Infection. *Cell Metab.* 29, 611-626.e5. (2019)

Comment 4:
Fig 3b, figure note errors?
Response: Thank you for pointing this out. The figure key and the corresponding figure 3b have now been corrected.

Comment 5:
An increase in LDs in the nuclei of WSSV-infected hemocytes at the viral late stage. Why the mitochondrial activity in WSSV-infected hemocytes was significantly increased at the late stage when the β-oxidation was inhibited?
Response: This is an interesting question – but we currently do not have a definitive answer. The best we can do, as noted in the discussion on lines 234-236, is to "speculate that the long duration of β-oxidation inhibition might have led to the activation of other energy pathways in order to maintain cell viability."

Comment 6:
The authors showed that blocking β-oxidation prevented further metabolic processing of the LCFAs induced by WSSV. Why the inhibition of β-oxidation consistently led to an increase in virion particles in both hemocytes and hemolymph?
Response: We too were surprised to find that viral genome copy numbers were unexpectedly increased in both hemocytes and hemolymph at the late stage. However, since the measurement was made on the viral genome copy numbers, and it is hard to distinguish a complete infectious virion from an incomplete virion, we speculate that the β-oxidation inhibition might somehow be promoting the premature release of the virion. Viral DNA might even be directly released into the hemolymph if the β-inhibition substantially disrupts the virion morphogenesis process. More work will need to be done to clarify whether β-oxidation inhibition can in fact cause the abnormal virion morphogenesis. We have now added a new sentence discussing this result (Lines 203-208)

"On the other hand, since our method for counting virion copy numbers would detect both complete and incomplete virions equally well, it is also possible that β-oxidation inhibition might be disrupting the process of virion morphogenesis in a way that causes large quantities of abnormal virions and/or naked viral DNA to be released into the hemolymph."

REVIEWERS' COMMENTS:

Reviewer #1 (Remarks to the Author):

The comments raised by this reviewer have been fully completed by the authtors.

Responses to the reviewers' comments:

Reviewer #1 .

Reviewer #1 (Remarks to the Author):

The comments raised by this reviewer have been fully completed by the authors.
Response: We are grateful for the reviewer's support and constructive feedback, which have helped us to strengthen the methodology and interpretation of our study.
